# Carbon Monoxide Prevents Hypertension and Proteinuria in an Adenovirus sFlt-1 Preeclampsia-Like Mouse Model

**DOI:** 10.1371/journal.pone.0106502

**Published:** 2014-09-09

**Authors:** Carolina C. Venditti, Richard Casselman, Iain Young, S. Ananth Karumanchi, Graeme N. Smith

**Affiliations:** 1 Department of Biomedical and Molecular Science, Queen's University, Kingston, Canada; 2 Department of Health Sciences, Queen's University, Kingston, Canada; 3 Department of Obstetrics and Gynecology, Kingston General Hospital, Kingston, Canada; 4 Department of Medicine, Beth Israel Deaconess Medical Center, Boston, Massachusetts, United States of America; VU University Medical Center, Netherlands

## Abstract

Preeclampsia (PE) remains a leading cause of maternal and neonatal morbidity and mortality worldwide. Smoking cigarettes is associated with a decreased incidence of PE. Based on this observation and previous work, we hypothesize that women who smoke have a lower risk of developing PE because of elevated levels of carbon monoxide (CO) in their blood. The objective of this study was to determine if low-dose CO in ambient air could attenuate the late pregnancy hypertension (HTN) and proteinuria in the Adenovirus (Ad) sFlt-1 PE-like mouse model. Continuous low-dose CO treatment (250 ppm) was started on E10.5 and maintained until E17.5. Compared to control and Ad empty vector, AdsFlt-1 mice displayed late- gestation HTN (E14.5–17.5) (P<0.05), proteinuria (P<0.05) and reduced Bowman's space which were all prevented with CO treatment. Use of the Ad (with/without sFlt-1) or CO had no effect (p>0.05) on litter size, fetal resorption numbers and fetal or placental weights. This study shows that treatment with CO can prevent HTN and proteinuria in a mouse model of PE. It provides a possible mechanism for the reduced incidence of PE in smoking women, and supports the possibility of using CO as a future treatment for PE.

## Introduction

Preeclampsia (PE) is a serious condition of pregnancy which presents as proteinuria and hypertension (HTN) [1], and is associated with underlying maternal vascular dysfunction [2]. There is no known cure other than delivery of the feto- placental unit. PE is thought to originate in the placenta, as a result of impaired implantation, leading to poor placental perfusion and hypoxia [1]. The resulting ischemia increases placental apoptosis and subsequent shedding of placental debris [3] and soluble factors into the maternal circulation, which are thought to contribute to maternal endothelial dysfunction and the clinical signs of PE [4]. Anti-angiogenic molecules are among these factors, as their overproduction leads to an imbalanced angiogenesis and is thought to contribute to the etiology of PE [5]. The best characterized anti- angiogenic molecule relating to PE is soluble *fms*- like tyrosine kinase-1 (sFlt-1), an alternatively spliced, soluble form of the VEGF receptor 1 [6]. It has been measured at increased levels in the placenta [7], amniotic fluid [8] and maternal serum [7] of women with PE. As an anti- angiogenic protein, sFlt-1 binds to the angiogenic VEGF and placental growth factor (PGF), rendering them inactive [9]. Both free VEGF and PGF are decreased in the circulation of women with PE [7].

There is no spontaneous animal model of PE and as a result, it has been difficult to determine the pathophysiologic process leading to the development and progression of this disorder. As well, it has limited our ability to develop therapeutic options for the prevention or treatment of PE. Using the association between PE and an imbalance of angiogenic factors, rodent models with PE-like signs (HTN and proteinuria) have been developed through the introduction and over production of sFlt-1 by adenovirus (Ad) [7], [10]–[12]. All studies report that late- stage HTN, proteinuria and glomerular damage ensue following the infection with AdsFlt-1 [7], [10]–[12].

A 33% reduced incidence in the development of PE has been associated with smoking cigarettes in pregnancy [13], [14] and in a dose dependent manner [15]. The same is not true of smokeless tobacco [15], [16], which led us to hypothesize that carbon monoxide (CO) a combustible product in cigarettes, was the agent conferring the reduction of PE [17]. Indeed, it has been shown that women with PE have reduced end- tidal breath CO levels [18]. Further, a negative correlation was determined between increased environmental ambient CO and PE [19].

We have previously demonstrated *in vitro* that CO is capable of reducing apoptosis in placental villous explants [20] and inducing vasodilation in the isolated perfused placenta [21] at concentrations similar to those in women who smoke during pregnancy. Further, *in vivo*, we have identified a dose of CO (250 ppm) that can be delivered to maternal mice without fetal gross morphological or developmental detriments, and which leads to maternal levels of CO similar to those of smoking women [22]. We have also shown that maternal mouse exposure to this dose of CO results in an increase of utero-placental blood flow and vascularity of the placenta [23]. Therefore, we hypothesized that the use of CO would prevent the development of HTN and proteinuria in the AdsFlt-1 rodent model of PE.

## Methods

### Ad replication

An Ad encoding Flt-1 (AdsFlt-1) (1–3) (first generation, E1 and E3 deleted) and Ad empty vector (AdEV) (Vector Labs, CA) were used to create a PE- like animal model as described previously [7], [24], [25]. Replication, virion concentration and replication competence were all carried out in our laboratory as described elsewhere [10]. We used a plaque assay [26] to determine infectious virus concentration. We performed a dose ranging study of sFlt-1 Ad and identified a dose of 1.25×10^8^ as the optimal dose that led to significant HTN and proteinuria without any dramatic fetal loss (see Table 1). Experimental groups of mice in either the AdsFlt-1 or AdEV group were injected at a dose of 1.25×10^8^ PFU infectious virions at E7.5.

**Table 1:** Comparison of maternal plasma sFlt-1 concentration and percentage of fetal loss for mice injected with high to low AdsFlt-1 concentrations.

AdsFlt-1 virion concentration for injection (PFU)	Number of mice injected with the AdsFlt-1	Maternal plasma sFlt-1 concentration (ng/ml ± SEM)	Percentage of litters that made it to term
1×10^9^	6	17 974±2979	0
5×10^8^	4	5456±962	0
2.5×10^8^	8	1262±153	57
1.25×10^8^	18	234±84	100

### Animals

All experimental procedures were approved by the Queen's University Animal Care Ethics committee (Smith-2012-003-Or-A2). Female CD-1 mice (8–10 weeks old), from Charles River Laboratories (Wilmington, MA), were provided with food and water *ad libitum*. Females were mated with males of the same strain overnight and the morning detection of a copulation plug was deemed E0.5. Females were weighed prior to mating and daily throughout the experiment.

### Treatment

Animals were separated into six groups: Control ± CO, AdEV ± CO and AdsFlt-1 ± CO. On E7.5, mice in the AdEV or AdsFlt-1 groups were injected with 100 ul of 1.25×10^8^ PFU via their tail vein. They were maintained in a biohazard room for 72 hrs and then moved back into our containment room. In order to eliminate effects of the Ad itself, we included the “control ± CO” group of mice who did not receive any Ad injections. Animals were sacrificed on E17.5 using 100 mg/kg of sodium pentobarbital intraperitoneally (Ceva Sante Animale, Libourne, France).

### Blood Pressure (BP) measurement and CO exposure

Prior to mating, each mouse was trained daily for two weeks using the Kent Scientific Tail cuff BP device (Connecticut, USA), ensuring a consistent BP reading was maintained over at least three consecutive days. Mice were then mated and BP was measured on each morning until E17.5. The mean of >5 stable and consistent measurements was taken as a true reading. BP was not measured on E8.5 -10.5, as mice were maintained in a biohazard room following their Ad injection. On E10.5 of pregnancy, the group of mice to be exposed to 250 ppm CO in ambient air were placed in a dosing chamber until E17.5, where CO levels were monitored continuously as previously described [22]. These mice were removed from the CO chamber for the daily BP measurements (∼20 min).

### Urine protein: creatinine concentration

Urine was collected at baseline (E0.5-1.5) and prior to sacrifice. Mice were placed in separate sterile cages lined with sterile 96 well plates, from which urine was collected and centrifuged at 4000×g for 10 min. The supernatant was removed and stored at −80°C. Urine was diluted 1∶2 in distilled water and protein concentration was measured using a Bradford assay (Quickstart Bradford assay, Biorad Mississauga, Canada). Urine was diluted 1∶10 in distilled water and creatinine was measured using a standard picrate method (Cayman Chemicals, Michigan, USA).

### Maternal blood collection and tests

Maternal blood was collected at early gestation as a baseline via the submandibular vein and prior to sacrifice via the retro- orbital vein. Blood CO measurements were performed using gas chromatography as previously described [22] and presented as percent carboxyhemoglobin (%COHb). Blood was centrifuged at 4000×g at 4°C for 20 min and the plasma was removed and stored at −80°C in aliquots. All plasma sFlt-1 concentrations were measured using a commercially available ELISA kit (MVR100, R&D systems, Minneapolis, MN); a 1/10 dilution (in calibrator diluent) was completed for all mouse baseline plasma, end of gestation plasma for Control ± CO and AdEV ± CO were diluted 1/10 in calibrator diluent and end of gestation plasma for AdsFlt-1 ± CO mice were diluted 1/100 in calibrator diluent.

### Procedures at time of sacrifice

Maternal mice were weighed prior to sacrifice. The maternal uterine horns were excised; litter number and fetal resorptions were noted. Un- resorbed fetuses and placentas were counted and weighed.

### Histology

At the time of sacrifice, mice were perfused with 4% PFA and harvested kidneys and placentas were placed in 4% PFA for 12 hours, paraffin embedded, sectioned at 3 µm and stained with H&E or periodic acid Schiff (PAS). Blinded renal H&E sections were imaged at 200X magnification; a minimum of 10 renal corpuscles per kidney were imaged. Using ImageJ software (ImageJ, U. S. National Institutes of Health, Bethesda, Maryland, USA), Bowman's capsule and glomeruli were measured in a blinded fashion and from these measurements the volume of the Bowman's space was calculated. The mean of 10 glomeruli measurements per mouse were compared and a minimum of 3 mice per group were used for comparison.

### Statistics

Statistical analysis was performed using Graphpad prism v5.0. All data is represented as means ± SEM. Mean daily systolic BP was calculated for each mouse, and these measurements were compared by two-way ANOVA with a post-hoc Bonferroni test. All other data was analyzed using a one way ANOVA with a post- hoc Tukey test. A probability value less than 0.05 was considered significant.

## Results

**Figure 1:**
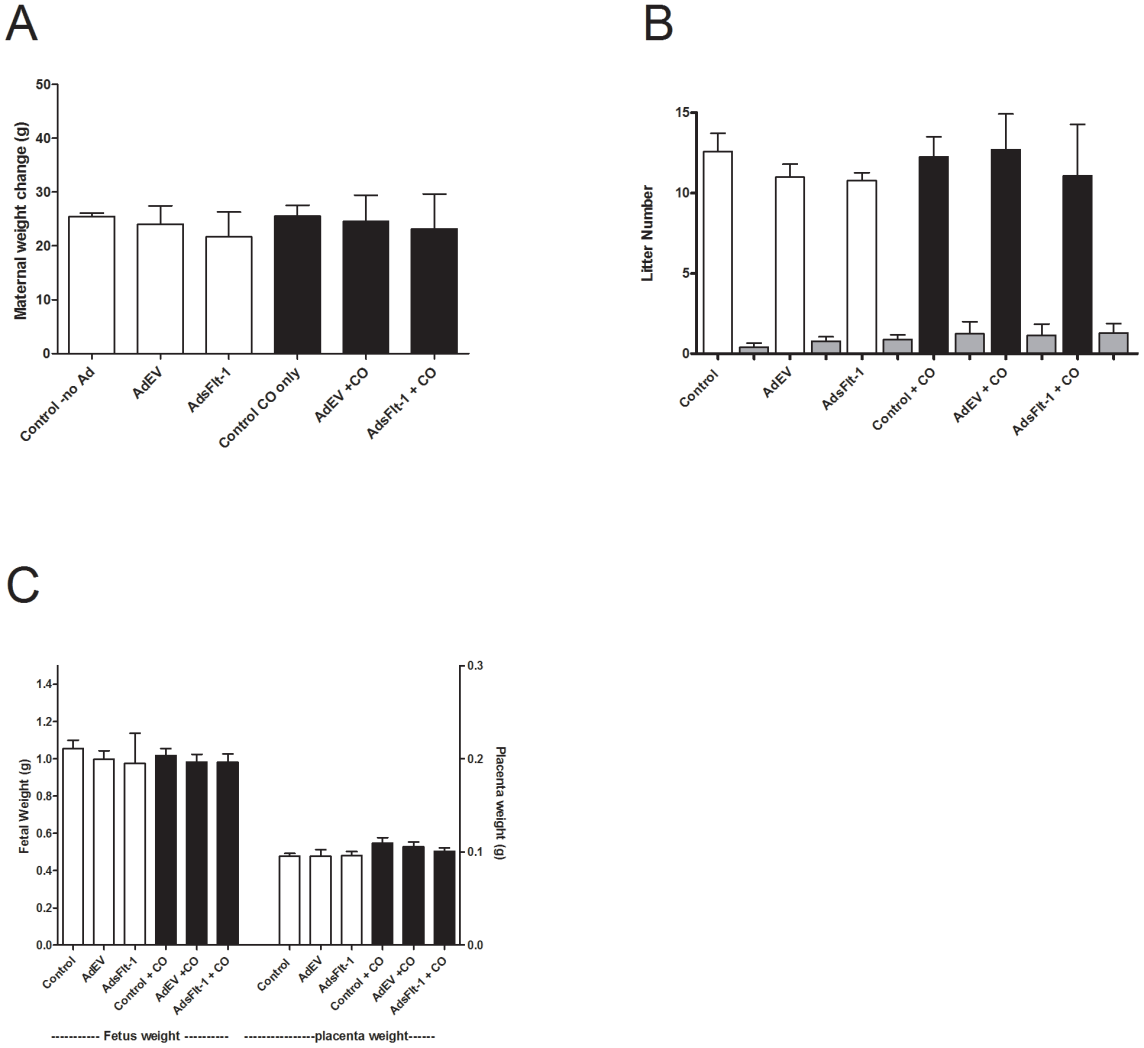
Maternal and fetal health were not negatively affected due to adenovirus (Ad) injection or CO exposure (black bars). A) Maternal change in weight throughout pregnancy was similar between all groups injected with Ad and exposed to CO (P>0.05). B) No change in litter size, or resorptions (grey bars) were observed between any of the six groups of mice. C) Fetal and placental weight were similar amongst all groups of mice, with no difference due to Ad or CO exposure (P>0.05).

Assessment of maternal weight change over gestation (weight prior to mating subtracted from weight at sacrifice) was used as a measure of health and this was not different between any of the six groups (Figure 1A). No differences between groups were noted in litter size or fetal resorption numbers (Figure 1B) or fetal/placental weight at birth (Figure 1C). Maternal blood CO levels (%COHb ± SEM) were consistent between Control, Ad empty vector (EV) and AdsFlt-1 groups not exposed to CO (Table 2). Further, maternal blood CO levels were not different between those mice exposed to CO, Control + CO, AdEV + CO and AdsFlt-1 + CO (Table 2). Mouse containment box CO concentrations were confirmed to be continuously delivering (ppm ± SD) 251.0±2.5 for all groups exposed to CO.

**Table 2:** Comparison of maternal blood carbon monoxide levels between groups of mice.

	%COHb ± SEM	Statistical Comparison
Control	0.61±0.85	a
AdEV	0.55±0.04	a
AdsFlt-1	0.54±0.01	a
Control + CO	9.93±1.06	b
AdEV + CO	10.63±3.57	b
AdsFlt-1 + CO	9.90±3.26	b

Similar letters denote no difference between groups, using a p-value of 0.05.

**Figure 2:**
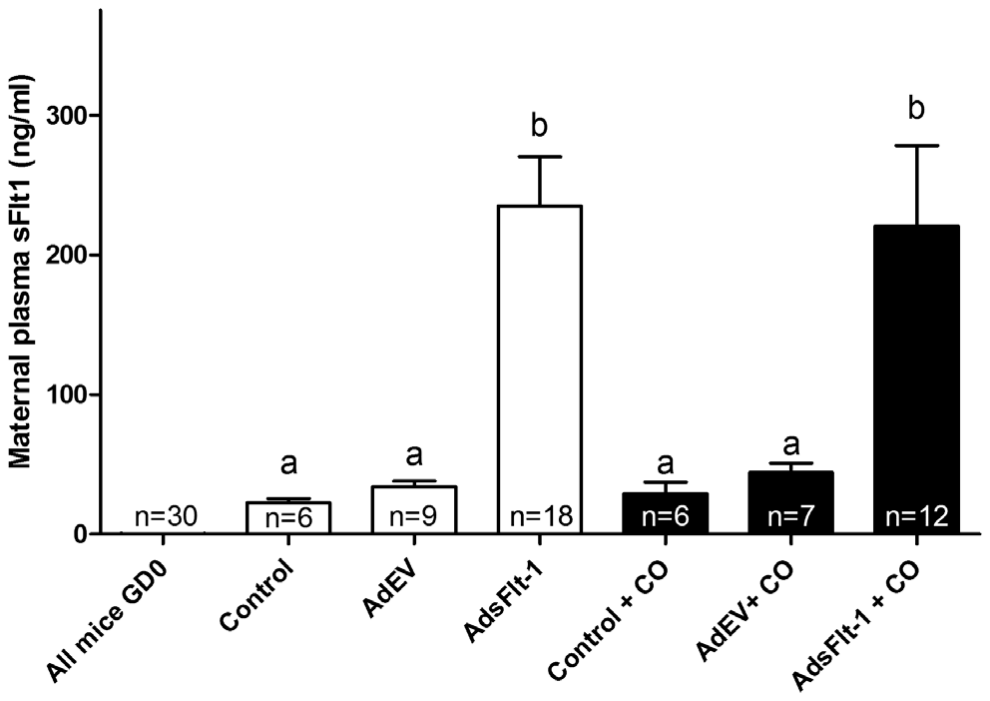
Tail-vein injection of AdsFlt-1 significantly increased maternal plasma sFlt-1 levels compared to control and AdEV groups. All maternal mouse sFlt-1 plasma levels (ng/ml ± SEM) were 0±1.23 at GD0.5 of pregnancy, but at term, both control and AdEV sFlt-1 levels were increased to levels observed in normal pregnancy. Mice injected with AdsFlt-1 measured with significant increases in plasma sFlt-1 levels, and this was not difference when exposed to CO. Similar letters represent data that is not statistically different.

Pregnant animals in each of the control and AdEV groups displayed increases in maternal plasma sFlt-1 concentrations (ng/ml ± SEM) from 0±1.23 at E0.5 to 22.39±6.46 and 33.82±11.65, respectively, at E17.5 (Figure 2). AdsFlt-1 injection resulted in sFlt-1 concentrations of 234.82±84.2, which was significantly different (P<0.05) from control and AdEV mice. CO exposure did not alter the sFlt-1 plasma concentrations (P>0.05) when compared to each respective group without CO exposure (Figure 2).

**Figure 3:**
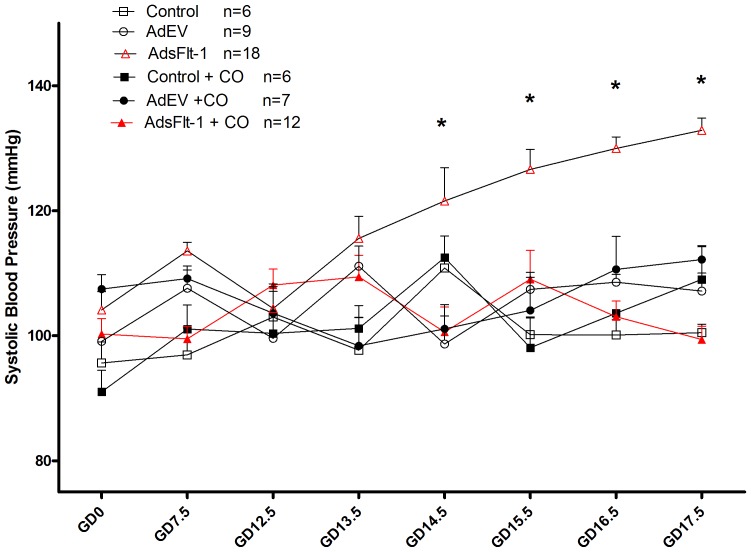
Maternal sFlt-1 –induced hypertension (HTN) in late stage pregnancy is completely normalized when mice are exposed to CO. No difference in BP throughout pregnancy was noted in control ± CO or AdEV ± CO groups throughout pregnancy. The injection of AdsFlt-1 led to HTN at the end of pregnancy (P<0.05), which was completed attenuated in mice exposed to CO and not different from control or AdEV groups (P>0.05).

Control mice and those administered AdEV displayed similar BP with no change across gestation (Figure 3). Treatment with CO in these groups did not affect the BP (P>0.05). Mice injected with AdsFlt-1 displayed late- stage HTN, with significant differences from each of control ± CO and AdEV ± CO (P<0.05) on E14.5 through 17.5. Treatment of this group with CO completely prevented (P<0.05) the HTN (Figure 3).

**Figure 4:**
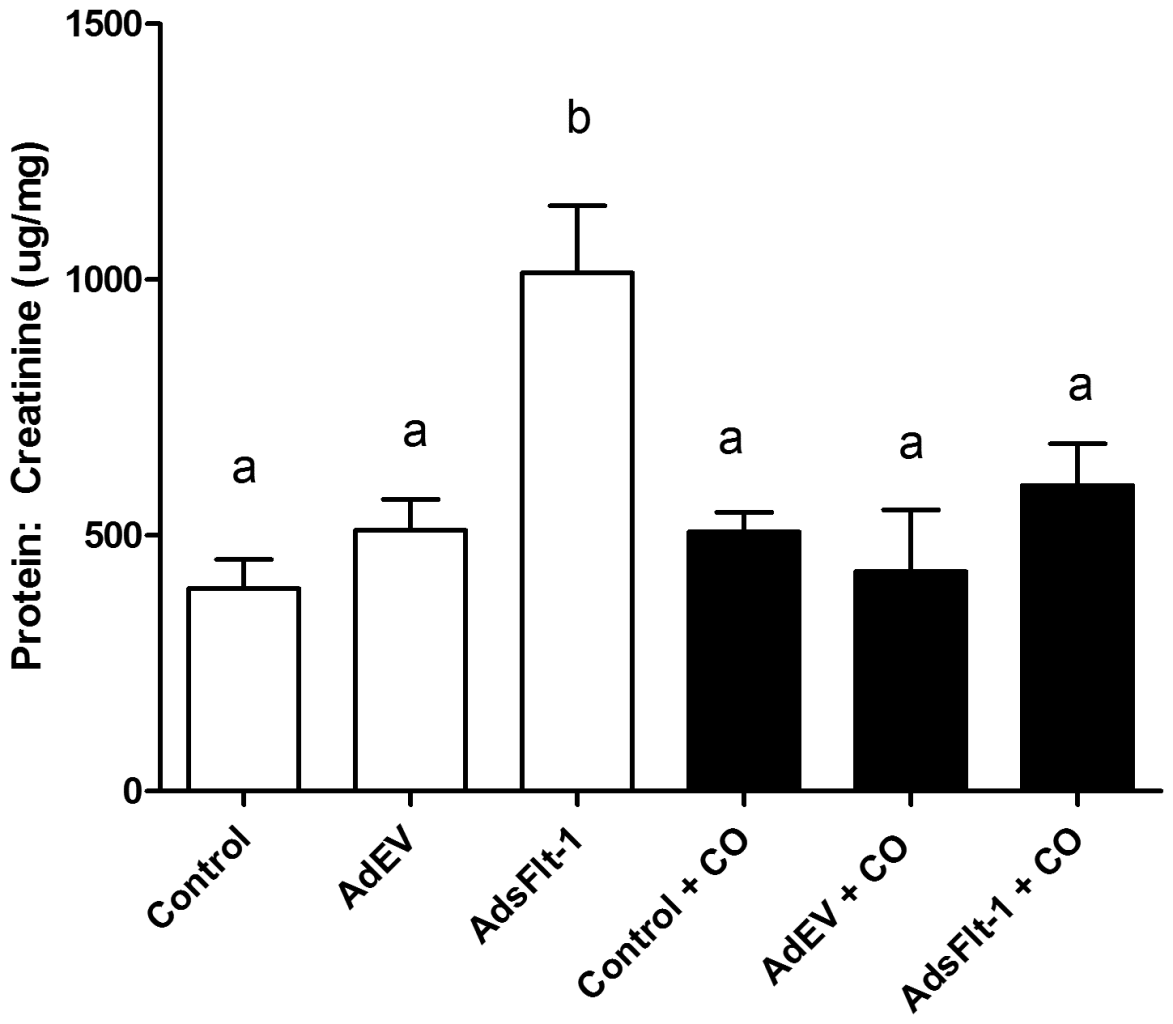
AdsFlt-1 injection leads to maternal proteinuria and is attenuated in mice exposed to CO. Mice injected with AdsFlt-1 displayed significant increases (P<0.05) in urine protein: creatinine ratios as compared to control and AdEV groups of mice. Exposure to CO reduced proteinuria levels to those of mice in both control ± CO and AdEV ± CO groups.

Late gestation urine protein: creatinine ratios were significantly increased in AdsFlt-1 injected mice (µg/mg ± SEM) (1013.0±135.4) compared to control (396.0±56.85) and AdEV (509.9±60.5) (Figure 4). When treated with CO, AdsFlt-1 mice were found to have reduced urine protein: creatinine ratios (µg/mg ± SEM) (597.8±81.0) compared to the AdsFlt-1 injected mice without CO treatment, (P<0.05). Mice treated with CO in each of control (506.82±270.6) and AdEV (428.96±292.16) had urine protein: creatinine (µg/mg ± SEM) levels similar to their respective groups without CO (P>0.05).

**Figure 5:**
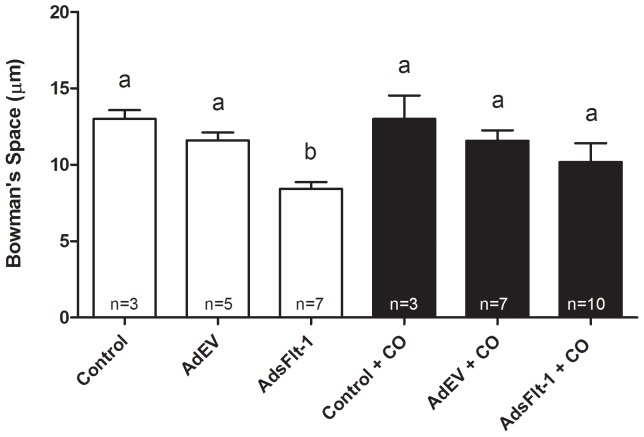
Bowman's space was significantly reduced in mice injected with AdsFlt-1 in comparison to control mice. No difference was observed in the Bowman's space between control ± CO and AdEV ± CO groups, as denoted by the letter a. Mice injected with AdsFlt-1 displayed significantly reduced renal Bowman's space, in comparison to control mice, denoted by the letter b. Exposure to CO normalized the Bowman's space, so that it was not different (P>0.05) than control ± CO, but was significantly increased (P<0.05) compared to mice injected with AdsFlt-1. The n- numbers displayed in the bar graphs represent the number of kidneys analyzed, where each kidney is a mean of 10 glomeruli measured.

Blinded analysis of the renal specimens by light microscopy displayed diffuse glomerular endotheliosis in mice injected with the higher doses of AdsFlt-1 (1×10^9^ PFU and 5×10^8^ PFU) (data not shown). At the virion concentration injected for the current study, blinded analysis did not identify differences for mice treated with AdsFlt-1± CO in comparison to all other groups (data not shown). However, the blinded measurement of Bowman's space revealed a decrease (P<0.05) in those mice injected with AdsFlt-1 (µm ± SEM) (8.43±1.0), in comparison to both control (13.0±2.7) and AdEV (11.6±1.15) mice (Figure 5). This was prevented (p<0.05) when AdsFlt-1 injected mice were treated with CO (11.2±2.35).

## Discussion

PE is a disorder unique to humans and as such, treatments are difficult to test, as adequate animal models of PE are few. One such animal model was created through the intravenous injection of AdsFlt-1 in the first half of rodent pregnancy [7]. This model increases maternal sFlt-1 protein levels, resulting in increased BP and altered renal histology and function, assessed through increased proteinuria [7]. George *et al*. reduced the sFlt-1– induced HTN in rats (non-pregnant), by injection of cobalt protoporphyin (CoPP), an inducer of heme oxygenase (HO) [27]. This endogenous enzyme functions to reduce the pro-oxidant free heme in the body by converting it into bilirubin, an antioxidant molecule [28], [29], free iron and CO, which at low levels has multiple physiologic functions [30]. *In vitro* studies using rat placental explant tissue have reported the reduction in hypoxia- induced sFlt-1 levels, through the induction of the HO system by administration of CoPP [31] or administration of CO- releasing molecules (CORMs) directly to placental villous explants [31]. Further, inhibition of HO-1 [by administration of Tin protoporphyrin (SnPP)] or HO-1 siRNA, leads to the augmentation of sFlt-1 release from the tissue. This data suggests that the properties of HO and its product CO could be used as a therapeutic direction to decrease sFlt-1 levels and normalize HTN and proteinuria, leading to a possible prevention or treatment of PE.

Our study used six different groups of mice, control ± CO and AdEV ± CO and AdsFlt-1 ± CO. In the control ± CO and AdEV ± CO groups, similar maternal plasma concentrations of sFlt-1 were found (P<0.05), indicating that the Ad itself and the CO treatment did not affect normal sFlt-1 production. In addition, levels of sFlt-1 in these four groups of mice were in- line with previously reported data for normal mouse pregnancy [10]. The group of mice injected with AdsFlt-1 had sFlt-1 protein concentrations (ng/mg ± SEM) (234.82±84.2) that were significantly increased (P<0.05) compared with AdEV (33.82±11.65) and control mice (22.39±6.46). These concentrations in the AdsFlt-1 injected mice were similar to published results in rats (215.5±81.2 ng/ml) [7] and CD-1 mice (87.7±4.5) [12]. It is worth noting that our AdsFlt-1 injection was lower than that published in each of the rat [7] or mouse [12] manuscripts, due to the different methodologies employed in measuring virus particles. Not surprisingly, addition of CO did not alter sFlt-1 concentrations in any of the AdsFlt-1 groups, as the exogenous addition of Ad travels to the liver of the animal and is continuously replicated, producing overexpression of the inserted construct, in this case, sFlt-1 [26].

The injection of AdsFlt-1 resulted in a significant increase in maternal BP in late- gestation compared to mice in the control and AdEV groups. Similar results for hypertensive effects of sFlt-1 injection have been reported in rats [7], [25], [27] and mice [10], [12], [24]. CO treatment prevented the maternal sFlt-1- induced HTN (Figure 3), while there was no effect on the AdEV or control groups BP. These findings are corroborated by results shown in rats, following the injection of an HO-1 inducer, CoPP, which significantly reduced the mean arterial pressure induced by sFlt-1 infusion [27]. The HO-1 system has been increasingly studied for the treatment of HTN in numerous forms of the disorder [32]–[35], and HO-1 likely acts in part through the vasodilatory function of its product CO [36]. While maternal sFlt-1 levels were not reduced, the prevention of HTN with CO treatment indicates that this action is likely independent of an effect CO might have on sFlt-1 production normally.

Glomerular endotheliosis is one of the hallmarks of PE [37], [38], leading to improper filtration and increased protein in the urine. Though several studies have indicated the clear late- pregnancy development of this renal pathology following treatment of animals with AdsFlt-1 [7], [10], [12], [25], our study did not reveal consistent glomerular histopathology, when viewed in a blinded fashion. However, injections of higher virion concentrations of AdsFlt-1 in pregnant mice led to diffuse glomerular endotheliosis, which was clearly evident when reviewed in a blinded fashion (data not shown). The measurement of Bowman's space demonstrated a significant reduction in the AdsFlt-1 group of mice (P<0.05), which was prevented by treatment with CO in comparison to the control and AdEV groups (Figure 5). Orsolic *et al.* [39] reported reduction in Bowman's space in mice with diabetic nephropathy and hypothesized that a reduction in capillary surface area available for filtration could have contributed to the development of proteinuria [40]. In the present study, urine protein levels were significantly increased in mice injected with sFlt-1 (Figure 4) which was prevented with CO treatment. It is possible that CO prevents the renal dysfunction induced by AdsFlt-1 at the level of the glomerulus, but it may also function through multiple different mechanisms, such as improvement of renal perfusion.

No difference was observed in in the maternal weight change, litter size and fetal resorption numbers with any of the treatments (AdEV, AdsFlt-1, CO) which we used to determine the effect on maternal or fetal health.

Manipulation of the HO/CO system is a possible therapeutic target for the prevention or treatment of PE. Women with PE have decreased levels of HO in their placentas, even prior to the development of the HTN and proteinuria [41]. In addition, they have reduced end- tidal breath CO levels [42], indicating a role for HO/CO in the development of PE. We have shown that exposure to low- dose CO can increase perfusion in the isolated perfused human placenta [21] and decrease apoptosis secondary to hypoxia/reoxygenation injury in placental explants [20]. Other research groups have shown that induction of HO-1 (by CoPP) leads to decreased placental apoptosis, potentially through the increased expression of Bag-1 at the feto- maternal interface [43]. These findings offer possible mechanisms for the HO/CO system to function at the level of the placenta and affect some of the findings associated with PE. Further, we have demonstrated that exposure of mice to 250 ppm CO throughout pregnancy increases both blood flow and angiogenesis of the uteroplacental unit [23]. Others have shown that treatment with 50 ppm CO in early gestation can reduce the incidence of miscarriage in an abortion- prone mouse model [44]. These studies are only a few of many which have attempted to explain how the HO/CO system could be implicated in complications of pregnancy, and certainly show positive results in the beneficial use of this system in the treatment of pregnancy complications, like PE.

This study shows that maternal low-dose treatment with CO can prevent the HTN and proteinuria in a PE-like mouse model induced by AdsFlt-1 injection. Future studies should focus on the mechanisms by which CO improves PE signs and symptoms in the sFlt-1 overexpression model. A prevention or treatment for PE remains elusive to date, but this study suggests that targeting the HO/CO system offers a promising direction towards a possible therapeutic for PE.
